# Supplementary material for: Multithreaded variant calling in elPrep 5
Source: PLoS One. 2021 Feb 4;16(2):e0244471. doi: 10.1371/journal.pone.0244471 (PMC7861424; doi:10.1371/journal.pone.0244471)
Supplement: S1 Appendix — We describe in detail how we reformulated the haplotype caller algorithm from GATK4 as a parallel algorithm that fits into the elPrep 5 framework, as well as the numerous challenges we faced for guaranteeing identical output to GATK4. (PDF) [file pone.0244471.s001.pdf]

S1 Appendix: Haplotype caller in elPrep 5

Charlotte Herzeel[1◑*], Pascal Costanza[1◑], Dries Decap[1,2], Jan Fostier[1,2], Roel Wuyts[1], Wilfried Verachtert[1]

1 ExaScience Life Lab, imec, Leuven, Belgium
2 Department of Information Technology, Ghent University - imec, Ghent, Belgium

◑These authors contributed equally to this work.
* Charlotte.Herzeel@imec.be

Structure of the haplotype caller in GATK 4

The haplotype caller algorithm for variant calling performs several computations on aligned sequencing data, and is far more involved than any of the other processing steps that we have implemented in earlier versions of elPrep.

The GATK 4 implementation structures the execution of the algorithm in the following way:

1. The purpose of the first stage is to direct the rest of the algorithm which locations in the reference and which reads in the input BAM file to process. This is achieved by an outermost loop that iterates over coarse-grained intervals, either as provided by the user, for example in the form of a BED file, or by simply iterating over the contigs as indicated in the header of the input BAM file. This results in a sequence of intervals or contigs, and the rest of the algorithm is applied to each element of this sequence, one by one. Alignment data is not yet fetched from the input BAM file at this stage.

2. The second stage determines a number of *assembly regions* for each interval or contig. An *active* assembly region is a short genomic region in which alignments sufficiently deviate from the reference, which indicates that there are one or more actual variants present. To ensure that variant calling is computationally feasible (see below), the haplotype caller algorithm by default restricts the maximum size of each assembly region to 300 base pairs. Assembly regions of maximum size 300 base pairs in between active regions are also passed to the next steps as *inactive* assembly regions, since the haplotype caller also computes reference confidence values for inactive regions when executing in GVCF or BP_RESULOTION mode.

   Assembly regions are generated by an iterator object, which is internally structured in the following way:

   (a) A pileup iterator fetches alignments from the BAM file input, and collects all reads that cover each position in the reference in ascending order. This iterator also implicitly applies a downsampling filter such that each reference position has a maximum number of alignments that start from this position. The exact maximum number can be configured as a command line parameter, and depending on its value, can even disable downsampling. However, the default behavior of GATK is to downsample down to 50 starting alignments per reference position.

(b) The assembly region iterator then internally builds activity profiles as a list of activity states computed per reference position, by fetching pileups from the above pileup iterator. Sublists of these activity states are dynamically split off based on parameters like activity thresholds, maximum assembly region size, and so on. These sublists are then combined into assembly region objects. When pileups sufficiently deviate from reference bases, the corresponding assembly region is marked as active, otherwise as inactive. These assembly region objects are returned by the assembly region iterator.

3. An inner loop iterates over the assembly regions fetched from the above assembly region iterator, and invokes the variant caller on each region. The variant caller proceeds as follows:

   (a) If the assembly region is inactive or empty, a reference model for no variation is computed and returned. (This check is repeated a few times throughout the variant caller due to modifications to the region that may effectively remove signs of variation.)

   (b) Otherwise, the reads that overlap, or are contained in, the assembly region are assembled using a De-Bruijn-like graph, which results in a number of candidate haplotypes.

   (c) For each candidate haplotype, its variation events are determined.

   (d) Based on these results, the assembly region is trimmed down to a potentially smaller region.

   (e) For the remaining region, the read likelihoods for each haplotype candidate are computed using a pair HMM algorithm. (This is computationally the most demanding step in the haplotype caller algorithm, which is why restricting the size of assembly regions further above is important.)

   (f) Based on this result, the reads are realigned.

   (g) This is followed by a genotyping step, producing the actual variant calls.

   (h) Finally, the reference confidence is computed both for reference positions with and without variant calls.

4. The result of each invocation of the variant caller on the assembly regions is finally written out to a VCF file. However, the variation information first needs to be combined depending on whether the haplotype caller executes in GVCF or BP_RESOLUTION mode (requiring the output of reference confidence values either as summary statistics for subregions, or for each reference position separately), or else in NONE mode (requiring no reference confidence values).

Due to the nested loops and iterators in the original expression of the haplotype caller in GATK 4, and the fact that reads are streamed from file input sequentially during the pileup creation step, the GATK implementation becomes inherently sequential. In "Intel" mode, step 3.(e) is parallelized, which is computationally the most demanding step, but the performance improvement is therefore also limited to this single substep in the overall algorithm.

Parallelization of the haplotype caller in elPrep 5

In order to design a parallel version of the overall haplotype caller algorithm, we have maintained the coarse-grained structure along the four main algorithmic blocks described above, but instead of employing loops and iterators, we have organized them

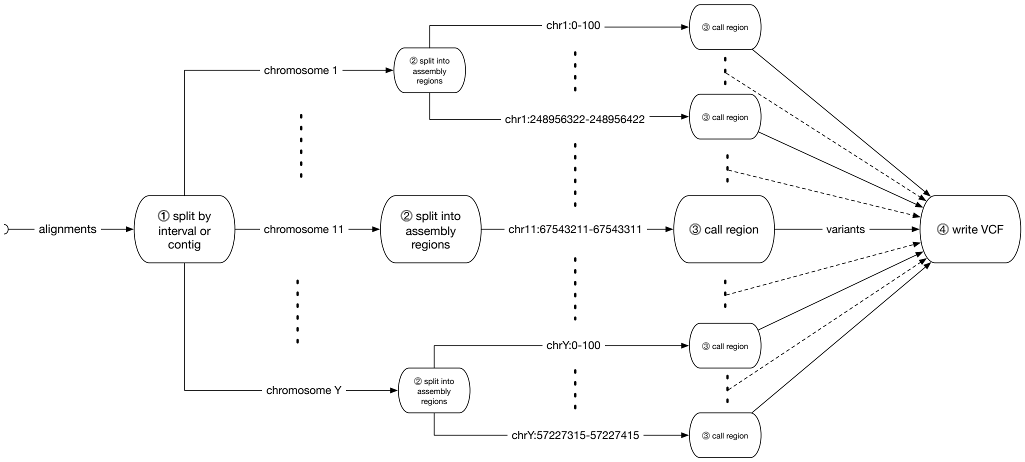

Fig 1. Parallelization of the haplotype caller in elPrep 5. Phase 1 groups reads by interval or contig, and hands over each group as soon as possible to phase 2. Phase 2 splits groups into assembly regions, and hands over each assembly region as soon as possible to phase 3. Phase 3 calls each region and hands over the variant calls to phase 4, which writes them to the result VCF file. All four phases can execute in parallel, and phase 2 and 3 can operate on multiple groups and assembly regions in parallel.

into a parallel pipeline architecture [3] consisting of four phases corresponding to these algorithm blocks.

The pipeline architecture described here is different from the parallel framework for the rest of elPrep: While elPrep so far allows for alternating between parallelizing over individual alignments and performing operations over the whole set of alignments, the haplotype caller algorithm executes operations over ranges of alignments that contain more than a single read, but are significantly smaller than the whole data set.

Fig 1 illustrates the four phases of elPrep's implementation of the haplotype caller. Parallel execution is achieved in three ways:

1. Phase 1 needs to group the alignments according to interval or contig boundaries and prepare them for further processing per group in the remaining phases. As soon as phase 1 completes the preparation for one interval or contig, it hands it over to phase 2, which enables phase 1 and 2 to operate in parallel: While phase 2 operates on one set of intervals or contigs, phase 1 can already prepare the next ones. This extends to all four phases which can all simultaneously operate on different sets of intervals or contigs.

2. In addition, phase 2 can operate on multiple intervals or contigs in parallel at the same time. The same holds for phase 3. In contrast, phases 1 and 4 are inherently sequential and can only operate on exactly one interval or contig each at the same time.

3. There are several computational steps within each phase which can be further parallelized locally, like for example the pair HMM algorithm.

In the following subsections, we describe the different phases of the haploytpe caller as implemented in elPrep 5 in more detail.

Phase 1: Split alignments by interval or by contig

While in the GATK implementation, downsampling is performed as part of building the pileups in step 2, we are performing downsampling already in phase 1. The reason for

this choice is as follows: If more than the specified maximum number of reads start at any given position, then the concrete choice of which reads are retained and which reads are discarded is governed by a single pseudo-random number generator. In order for elPrep to produce the same results as GATK, we have reimplemented the same random number generator, and as a consequence, this phase also has to remain strictly sequential to ensure that elPrep's implementation sees the same series of random numbers in the exact same order as GATK. Since phase 1 is already inherently sequential, it is better to perform downsampling here to not adversely impact parallelization opportunities in phase 2.

The split up of the alignment data into either intervals or contigs is also already performed in this phase, unlike in GATK. Since all alignment data is already present in RAM in the elPrep implementation after the preceding preparation steps, there is no reason to delay the grouping of read data.

Phase 2: Split each interval or contig into assembly regions

Phase 2 consists of the following steps:

1. Perform a pileup for each position in the reference.
2. Compute an activity probability for each position in the reference based on the corresponding pileup.
3. Determine the assembly regions based on these activity probabilities.

Steps 1 and 2 are embarrassingly parallel. Step 3 is sequential since the different active regions can be of different length, which means that each assembly region can only be computed when the previous assembly regions are already known in the current interval or contig.

Since alignments are potentially arbitrarily long in the reference due to hypothetically arbitrarily long deletions, the pileup algorithm in step 1 would, without further effort, have to check all alignments to the left of a given position if they overlap, even if they are very far away.

To avoid this costly repeated reexamination of each read for each reference position, we first compute the maximum reference length of all reads in a given contig, which is usually rather short. Determining this number can be efficiently implemented with a straightforward parallel reduction [3]. This number then helps to drastically reduce the reads to consider when determining the pileup for a particular reference position.

The assembly regions computed in this phase are finally handed over to phase 3, including both active and inactive regions like in GATK.

Phase 3: Perform variant calling on each assembly region

Variant calling is performed following the same steps as in GATK, i.e., by assembling reads using a De-Bruijn-like graph; computing read likelihoods using a pair HMM algorithm; realigning reads based on these likelihoods; genotyping the reads; and computing the reference confidence. Parallelism is achieved in this phase by expressing several steps in this phase as locally parallel algorithms, especially for computing event maps, pair HMM, realignment, and so on.

Phase 4: Write variant information to a VCF file

This phase is mostly sequential. However, just like for the rest of elPrep where the file representation of each entry in a SAM/BAM file is generated in parallel (either as a line

of string or as the corresponding binary representation), the text lines which represent VCF file entries are also generated in parallel.

Side channels

While in phase 3, the genotyping information for a variant that is part of an active region is computed, it is possible that some output alleles represent deletions that cover a stretch of the reference that is part of another active region further to the right. While computing the genotyping information for the latter active region, information from these output alleles from the former active region must be present. This means that, strictly speaking, there is unfortunately a sequential dependency between the different active regions.

To ensure that we can nevertheless perform variant calling on assembly regions in parallel, we add a "side channel" to each assembly region which can receive deletion information from other assembly regions to the left, and pass on deletion information to other assembly regions to the right. These side channels are properly synchronized, so can be safely accessed concurrently. Deletion information is passed to the right as early as possible, especially in the very common case when it can be known very early that no deletion information will be present in an assembly region. Likewise, deletion information is consumed from the left as late as possible. Deletion information can be efficiently passed through from an assembly region to the left to other assembly regions to the right if the current assembly region does not participate in the handling of deletion information (for example, for inactive regions).

The design of these side channels therefore ensures that variant calling can be performed as much as possible in parallel in spite of this rare sequential dependency.

Guaranteeing equivalent results

Our goal with elPrep has always been to produce results that are identical at the binary level to those of de-facto standard tools, like GATK [1,2]. This enables us to verify correctness of elPrep by performing straightforward comparisons using tools like Unix diff, without requiring a biological interpretation of the results.

As for the rest of elPrep, GATK's haplotype caller created some challenges in this regard which we had to resolve. We have based the elPrep implementation of the haplotype caller on version 4.1.3.0 of GATK. In the following subsections, we are discussing the challenges that we have discovered in that version of GATK, and our solutions.

Rounding modes for floating point operations

The Java and Go programming languages respectively use different rounding modes when performing floating point operations. For the haplotype caller, this can lead to results that are slightly different from each other, specifically because the log10 function may return slightly different results. We have solved this issue by providing our own log10 implementation in C with specific instructions to direct the CPU to use the same rounding mode as Java, and by calling that version from elPrep.

Formatting of floating point numbers

The Java and Go programming languages also differ with regard to how they print floating point numbers, specifically with regard to how many digits are printed after the comma. This can lead to different textual representations for the same floating point

values in the resulting VCF file, which makes it difficult to compare the results using Unix diff. We have solved this by providing our own implementation for formatting floating point numbers that mimics the Java behavior.

Non-determinism of De-Bruijn-like graphs

In the GATK implementation, version 4.1.3.0, when creating the De-Bruijn-like graphs, the order of how incoming and outgoing vertices of each node are stored is non-deterministic due to the use of a hashtable-based set implementation. This has an impact on the result of read assembly due to the resulting non-deterministic order in which nodes are visited during graph traversal. We have solved this issue by creating a modified version of GATK that uses a linked hashtable-based set implementation instead, which guarantees deterministic order, and by using a similar set implementation in elPrep by default. Later versions of GATK adopt the same linked hashtable-based set implementation for this purpose.

Random number generator

Furthermore, the Java and Go programming languages provide different random number generator algorithms. This has an impact in two areas of the haplotype caller algorithm:

1. By default, the haplotype caller limits the number of reads that start at a given reference position down to a specified maximum number. The choice which reads are retained and which reads are dropped is based on the outcome of the random number generator.
2. The haplotype caller algorithm computes a number of annotations to be included in the resulting VCF file during genotyping. Among others, this includes the "Quality by Depth" (QD) annotation. According to the code comments in the original GATK 4 code, the QD value can become unusually high when multiple events are on the same haplotype. To avoid that such results are filtered out by later tools (e.g., VQSR), the QD value is capped to a maximum value before it is being written out to the resulting VCF file. However, instead of simply setting QD to a fixed high value, the capping also adds Gaussian noise to the capped value, which is based on Java's random number generator.

The first issue is easily resolved by replacing Go's random number generator with our own implementation that mimics Java's algorithm and produces the exact same sequence of random numbers.

The second issue is more problematic: GATK reuses the same random number generator source for downsampling reads in the early stage as for capping QD values in the late stage. From a software engineering perspective, this is problematic, because in elPrep's parallel implementation of the haplotype caller it becomes non-deterministic which sequence of random numbers is seen for each purpose due to non-deterministic interleaving of operations of the several phases executing in parallel.

We have solved the second issue by creating a modified version of GATK's haplotype caller that caps the QD value to a fixed value, without adding any Gaussian noise. This means that the random number generator is not invoked for this purpose, so it is exclusively used only for downsampling. Likewise, we can compile a custom version of elPrep that removes the Gaussian noise from the capped QD value as well, producing then exactly the same result as GATK when using those two modified versions of GATK.

elPrep's "pedantic" and "fixed_high_qd" modes

The technical differences described above lead only to very minor differences in the resulting VCF files between the GATK and the elPrep implementations of the haplotype caller algorithm. It is therefore desirable to avoid using the workarounds described above by default to not incur unnecessary overheads (although they seem very small in practice).

More concretely, by default elPrep compiles with Go's default implementations of the log10 function, the formatting routines for floating point numbers, and its random number generator. To enable the workarounds discussed above for precise equivalence tests, elPrep has to be compiled in the so-called "pedantic" mode. In the "pedantic" mode, elPrep also ensures that the state of the random number generator is retained during processing of subsequent split files to guarantee equivalence even when using the sfm version of elPrep.

With regard to capping QD values, elPrep by default uses a separate random number generator source from the one used for downsampling. To disable adding Gaussian noise when capping QD values, elPrep has to be compiled in the so-called "fixed_high_qd" mode.

elPrep can be compiled both in "pedantic" and "fixed_high_qd" mode at the same time. See the elPrep documentation for details on how to compile elPrep in these different modes.

Summary

The algorithmic changes and challenges we described in the previous sections should give an idea of the complexity we faced when optimizing and integrating the GATK haplotype caller algorithm into elPrep. Parallelizing the execution of the haplotype caller algorithm is only one aspect for improving the performance, but we also had to simplify and optimize other algorithmic aspects. We estimate that we have touched around 33000 lines of Java code in GATK to synthesize the haplotype caller implementation in elPrep. Our haplotype caller code is around 9000 lines of Go code, but we left out GATK features like checking for sample contamination, ploidies other than diploid, and so on, which are not used in standard pipelines for processing human DNA data. On top of the 9000 lines of code that implement the core haplotype caller algorithm, we also have around 80000 lines of precomputed floating point values in the Go source code for certain mathematical operations that were precomputed in Java, so we can always obtain the same results in elPrep as in GATK.

References

1. Herzeel C, Costanza P, Decap D, Fostier J, Reumers J. elPrep: High-Performance Preparation of Sequence Alignment/Map Files for Variant Calling. PLoS ONE. 2015;10(7). doi:10.1371/journal.pone.0138868.

2. Herzeel C, Costanza P, Decap D, Fostier J, Verachtert W. elPrep 4: A multithreaded framework for sequence analysis. PLoS ONE. 2019:14(2). doi:10.1371/journal.pone.0209523.

3. McCool M, Robison AD, Reinders J. Structured Parallel Programming - Patterns for Efficient Computation. Waltham, Massachusetts: Morgan Kaufman; 2012.
