# Supplementary material for: Multithreaded variant calling in elPrep 5
Source: PLoS One. 2021 Feb 4;16(2):e0244471. doi: 10.1371/journal.pone.0244471 (PMC7861424; doi:10.1371/journal.pone.0244471)
Supplement: S2 Appendix — We discuss benchmarks that compare elPrep 4 and elPrep 5 in terms of runtime, peak memory use, and disk use, when executing a pipeline that consists of four steps to prepare data for variant calling. (PDF) [file pone.0244471.s002.pdf]

S2 Appendix: Benchmarks comparing elPrep 4 and elPrep 5

Charlotte Herzeel[1◑*], Pascal Costanza[1◑], Dries Decap[1,2], Jan Fostier[1,2], Roel Wuyts[1], Wilfried Verachtert[1]

1 ExaScience Life Lab, imec, Leuven, Belgium
2 Department of Information Technology, Ghent University - imec, Ghent, Belgium

◑These authors contributed equally to this work.
* Charlotte.Herzeel@imec.be

We set up an additional benchmark to compare the performance of elPrep 4 and elPrep 5 on the preparation steps of the pipeline. The goal is to see if our changes to the elPrep framework and processing engine for implementing variant calling have an impact on the performance of previously existing functionality.

Fig 1 shows a benchmark comparing GATK 4, elPrep 4, and elPrep 5 for executing a pipeline that consists of 4 preparation steps (sorting by coordinate order, duplicate marking, base quality score recalibration and application) on a whole-exome sample. We show graphs comparing runtime, RAM use, and disk use. When comparing the elPrep sfm mode in elPrep 4 and elPrep 5, we see only negligible differences, with both runs having a similar speedup (±8x), RAM use (±90%), and disk use (±50%) compared to GATK. For the elPrep filter mode, we see a small difference between speedup and RAM use compared to GATK, as we measure ±16x speedup and ±2.5x RAM use in elPrep 4, and ±14x speedup and ±3.5x RAM use in elPrep 5.

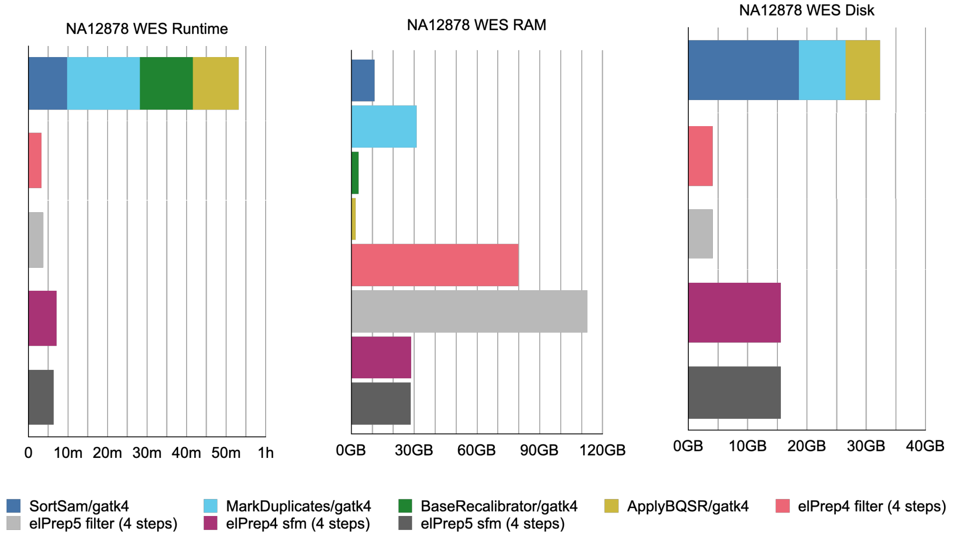

Fig 1. WES benchmarks elPrep 4 vs elPrep 5 For the elPrep sfm mode, we see no real differences between elPrep 4 and elPrep 5, which produce similar speedup and RAM and disk use compared to GATK 4. There is a difference between elPrep 4 and elPrep 5 for the filter mode. This is because elPrep 5 implements the GATK semantics of the `-L` option for operating on targeted regions, whereas elPrep 4 only had an option that implements the SAMtools `-L` semantics. These options differ slightly, specifically at which step of the execution reads from non targeted regions are filtered out.

The difference between elPrep 4 and elPrep 5 for the filter mode is because of a change we made to support variant calling on targeted regions so that we follow the GATK 4 semantics. Specifically, in elPrep 5, we add an option `--target-regions` for specifying which regions in the genome a pipeline should operate on. This option is equivalent to the GATK `-L` option that can be passed to the base quality score recalibration and haplotype caller tools. In elPrep 4, we have the `--filter-non-overlapping-regions` option that implements the SAMtools `-L` semantics, which is similar to, but not identical to, the GATK `-L` option. Specifically, the `--filter-non-overlapping-regions` option removes reads from the input *before* any of the pipeline steps are executed, while the `--target-regions` only does so *after* the base quality score recalibration step. Hence with the GATK `-L` semantics, the first steps in the pipeline operate on more read data. In the elPrep filter mode, this means more data is read into RAM, hence a higher peak is seen in the elPrep 5 run, creating a bit of additional overhead in terms of memory management and slowing down execution time a bit. This difference between elPrep 4 and elPrep 5 is mitigated when using the `--filter-non-overlapping-regions` option in elprep 5 as well, but this may result in slightly different results later down the analysis when performing variant calling using the haplotype caller algorithm.

Fig 2 shows the results for comparing elPrep 4 and elPrep 5 on a whole-genome data set. There are no significant differences, as both runs yield a similar speedup (±10x) and resource use (±70% of RAM and ±50% of disk use) compared to GATK.

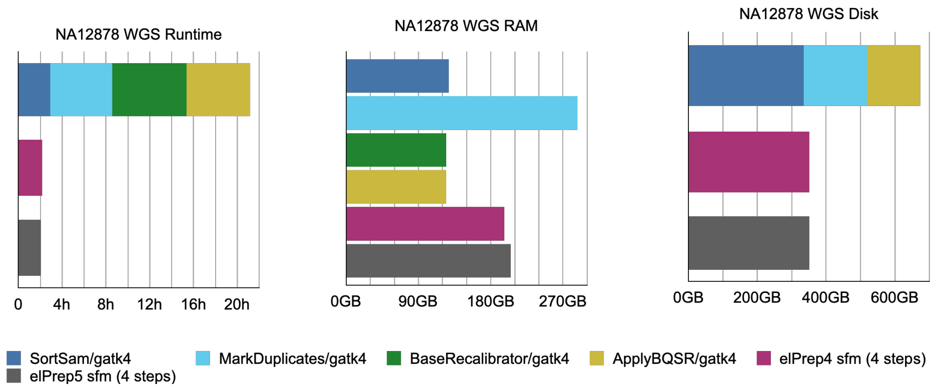

Fig 2. WGS benchmarks elPrep 4 versus elPrep 5 There is no significant difference between elPrep 4 and elPrep 5. Both runs have a ±10x speedup compared to the GATK run and use ±70% of the RAM and ±50% of the disk GATK uses.
